# Soluble Epoxide Hydrolase Inhibition Prevents Experimental Type 4 Cardiorenal Syndrome

**DOI:** 10.3389/fmolb.2020.604042

**Published:** 2021-03-11

**Authors:** Mouad Hamzaoui, Clothilde Roche, David Coquerel, Thomas Duflot, Valery Brunel, Paul Mulder, Vincent Richard, Jérémy Bellien, Dominique Guerrot

**Affiliations:** ^1^ Normandie University, UNIROUEN, INSERM U1096, FHU REMOD-VHF, Rouen, France; ^2^ Nephrology Department, Rouen University Hospital, Rouen, France; ^3^ Pharmacology Department, Rouen University Hospital, Rouen, France; ^4^ Biochemistry Department, Rouen University Hospital, Rouen, France

**Keywords:** cardiorenal syndrome, heart failure, endothelial (dys)function, chronic kidney disease, epoxyeicosatrienoic acid

## Abstract

**Objectives:** Cardiovascular diseases (CVD) remain the leading cause of morbimortality in patients with chronic kidney disease (CKD). The aim of this study was to assess the cardiovascular impact of the pharmacological inhibition of soluble epoxide hydrolase (sEH), which metabolizes the endothelium-derived vasodilatory and anti-inflammatory epoxyeicosatrienoic acids (EETs) to dihydroxyeicosatrienoic acid (DHETs), in the 5/6 nephrectomy (Nx) mouse model.

**Methods and Results:** Compared to sham-operated mice, there was decrease in EET-to-DHET ratio 3 months after surgery in vehicle-treated Nx mice but not in mice treated with the sEH inhibitor *t*-AUCB. Nx induced an increase in plasma creatinine and in urine albumin-to-creatinine ratio as well as the development of kidney histological lesions, all of which were not modified by *t*-AUCB. In addition, *t*-AUCB did not oppose Nx-induced blood pressure increase. However, *t-*AUCB prevented the development of cardiac hypertrophy and fibrosis induced by Nx, as well as normalized the echocardiographic indices of diastolic and systolic function. Moreover, the reduction in endothelium-dependent flow-mediated dilatation of isolated mesenteric arteries induced by Nx was blunted by *t*-AUCB without change in endothelium-independent dilatation to sodium nitroprusside.

**Conclusion:** Inhibition of sEH reduces the cardiac remodelling, and the diastolic and systolic dysfunctions associated with CKD. These beneficial effects may be mediated by the prevention of endothelial dysfunction, independent from kidney preservation and antihypertensor effect. Thus, inhibition of sEH holds a therapeutic potential in preventing type 4 cardiorenal syndrome.

## Introduction

Chronic kidney disease (CKD) is an important health care problem with a worldwide prevalence around 13% (Hill et al., 2016). CKD is an independent risk factor for cardiovascular (CV) diseases and CV events are the first cause of death in this population (Hatamizadeh et al., 2013). The association between CKD and the occurrence of chronic heart disease has been named uremic cardiopathy or type 4 cardiorenal syndrome (Zannad Faiez and Rossignol Patrick, 2018). In this setting, the crosstalk between the diseased kidney and heart is not fully understood. The pathophysiology of type 4 cardiorenal syndrome is predominantly mediated by reciprocal hemodynamic effects, neurohormonal activation, inflammation, anaemia, disturbances in the hydro electrolytic balance and abnormalities of the bone-mineral axis (Kovesdy and Quarles, 2013; Kovesdy and Quarles, 2016; Ter Maaten et al., 2016; Rangaswami et al., 2019). Fibroblast growth factor-23 (FGF-23), secreted by osteocytes, regulates phosphate and vitamin D homeostasis and its plasma concentration rises as the kidney function declines. Several studies have shown that higher levels of FGF-23 are associated with a higher risk of death (Scialla et al., 2014; Charytan et al., 2015).In spite of significant breakthroughs in our understanding and treatment of the CV disease associated with CKD, mortality remains high and identifying new therapeutic targets is of critical importance.

Epoxyeicosatrienoic acids (EETs) are arachidonic acid derivatives synthesized by cytochrome P450 (CYP450) epoxygenases (Wang Z. H. et al., 2013; Duflot et al., 2014). EETs are endothelium-derived hyperpolarizing factors with potent antihypertensive, anti-inflammatory and anti-proliferative properties. They are rapidly hydrated into the less active dihydroxyeicosatrienoic acids (DHETs), by an enzyme called soluble epoxide hydrolase (sEH). Thus, inhibition of sEH appears as an interesting pharmacological approach to increase the bioavailability of EETs in various pathological conditions in particular cardiovascular diseases (Wang Z. H. et al., 2013; Duflot et al., 2014). sEH inhibitors were shown to reduce hypertension and endothelial damage in angiotensin II-dependent models (Imig et al., 2002; Gao et al., 2011) and to prevent cardiac dysfunction and remodelling in experimental heart failure (Merabet et al., 2012). In human, genetic polymorphisms of *EPHX2,* which encodes sEH, could either increase hydrolase activity which is associated with a higher occurrence of ischemic cardiac events (Lee et al., 2006), or decrease its activity associated with higher endothelium-dependant dilatation in resistance arteries (Lee et al., 2011), strengthening the interest of inhibiting sEH in cardiovascular diseases. At the renal level, the beneficial impact of sEH has been reported in several works (Liu, 2018) but not all (Jung et al., 2010), and no study has evaluated the interest of this strategy in type 4 cardiorenal syndrome.

In this study we analysed the impact of sEH inhibition on the CV consequences induced by 5/6 nephrectomy (5/6 Nx) in mice, a classical model of CKD.

## Material and Methods

### Animals and Treatments

All experiments were carried out in 129/Sv male mice, aged 8 weeks and weighing between 20 and 26 g (January laboratory, Genest Isle), in accordance with the standards and ethical rules (CENOMEXA C2EA-54). The surgical procedures were performed by a single experienced operator in order to ensure reproducibility. Briefly, surgical procedure consisted in ligation of the upper branch of the left kidney artery, followed by a cauterization of the lower pole of the left kidney, leading to 2/3 of non-functioning left kidney. One week later, the right kidney was removed, inducing 5/6 Nx. One week after removal of the right kidney, 5/6 Nx mice were randomized into two groups to receive either the sEH inhibitor *trans*-4-[4-(3-adamantan-1-yl-ureido)-cyclohexyloxy]-benzoic acid (*t*-AUCB: 15 mg/L in drinking water after dilution in 15-ml PEG 400) or vehicle (PEG 400) until sacrifice. A third group of sham-operated mice (surgical laparotomy) served as controls.

### Blood and Urine Analyses

One month after surgery, 100 µL retro-orbital blood samples were collected in anesthetized animals (isoflurane: 1.5%) to quantify plasma creatinine by enzymatic method. At sacrifice, blood samples were collected allowing to measure plasma creatinine, soluble vascular cell adhesion molecule 1 (sVCAM-1) and fibroblast growth factor-23 (FGF-23) were assessed by enzyme-linked immunosorbent assays (ab100750, Abcam and EZMFGF23–43K, EMD Millipore respectively). In addition, the plasma levels of 14,15-EET, the preferential substrate of sEH, its metabolite 14,15-DHET, and the pro-inflammatory hydroxyeicosatetraenoic acids (5-HETE, 12-HETE and 15-HETE), derived from the lipoxygenase (LOX) metabolites of arachidonic acid, were quantified by LC-MS/MS using a previously published method (Duflot et al., 2017; Duflot et al., 2019). The ratio of 14,15-DHET-to-14,15-EET was used as an index of sEH activity.

24-h urine was collected 1 and 3 months after surgical procedure using metabolic cages. Urine albumin and aldosterone were measured at 3 months using enzyme-linked immunosorbent assays (ab108792 and ab136933, respectively; Abcam, Paris, France). Na^+^ excretion was quantified using standard procedure.

### Systolic Blood Pressure

Non-invasive measurements of systolic blood pressure were performed by tail cuff plethysmography (CODA, Kent Scientific Corporation) 1 and 3 months after surgery. These measurements were performed in conscious and trained mice and consisted in two series of 10 cycles of measurements for each animal.

### Cardiac Function

In mice anesthetized with isoflurane (1.5–2%), left ventricular end-diastolic and end-systolic diameters (LVEDD and LVESD, respectively) were assessed 1 and 3 months after surgery, using a Vivid seven ultrasound device (GE medical). The heart was imaged in the two-dimensional mode in the parasternal short-axis view. Ejection fraction (EF) was calculated from the LV cross-sectional area as EF(%) = ((LVDA – LVSA)/LVDA) x 100 where LVDA and LVSA are LV diastolic and systolic area, estimated from LVEDD and LVESD. In addition, a pulsed Doppler of the LV outflow was performed to obtain heart rate (HR). Doppler measurements were made at the tip of the mitral leaflets for diastolic filling profiles in the apical four-chamber view, allowing to determine peak early (E) and late (A) mitral inflow velocities, and calculation of the E/A ratio, as estimate of diastolic function.

### Vascular Function

Flow-mediated dilatation (FMD) was assessed on second mesenteric resistance artery segment. Briefly, the mesentery was removed and placed in cold oxygenated Krebs buffer. A 2–3 mm segment of third mesenteric resistance artery segment was isolated and mounted on an arteriograph (DMT, Denmark). Vessels were pre-constricted using 10^-5^ M phenylephrine (Phe) before assessing the dilatory response to stepwise increase in intraluminal flow (3, 6, 10, 15, 20, 25, 50, 75 and 100 μL/min). FMD was assessed in the absence and in the presence of the NOS inhibitor Nω-nitro-l-arginine (l-NAME; 10–4 M) and the CYP450 epoxygenase inhibitor Fluconazole (10–4 M).

### Histology

In 5/6 Nx mice, the remnant kidney was removed at sacrifice. Kidney histological lesions were analysed after Masson's staining as previously described (Guerrot et al., 2011). The slides were independently examined on a blinded basis, using a 0 to four injury scale for the level of interstitial inflammation, interstitial fibrosis and glomerulosclerosis at magnification x20 (0: no damage; 1: <25% of kidney damaged; 2: 25–50% of kidney damaged; 3: 50–75% of kidney damaged; 4: 75–100% of kidney damaged). Tubular lesions were analysed at magnification x10. Vascular thickening and vascular fibrosis were analysed at magnification x40. The upper half of kidney was not analysed since it was ischemic in 5/6 Nx groups.

The heart was harvested, weighed and a section of the left ventricle was snap-frozen for subsequent determination of LV fibrosis, using 8-µm thick histological slices stained with Sirius Red as previously described (Guerrot et al., 2011).

### Statistical Analyses

Data were expressed as mean values ± SEM. Comparison between groups was performed by one-way ANOVA, followed by Tukey multiple comparison post-tests, For FMD, analysis was performed by two-way ANOVA with Dunnett’s multiple comparisons test. A value of *p* < 0.05 was considered statistically significant. Survival was analysed using Log-rank (Mantel-Cox) test. Statistical tests were performed with Prism software version 8 (8.0.2 263).

## Results

### sEH Activity

**FIGURE 1:**
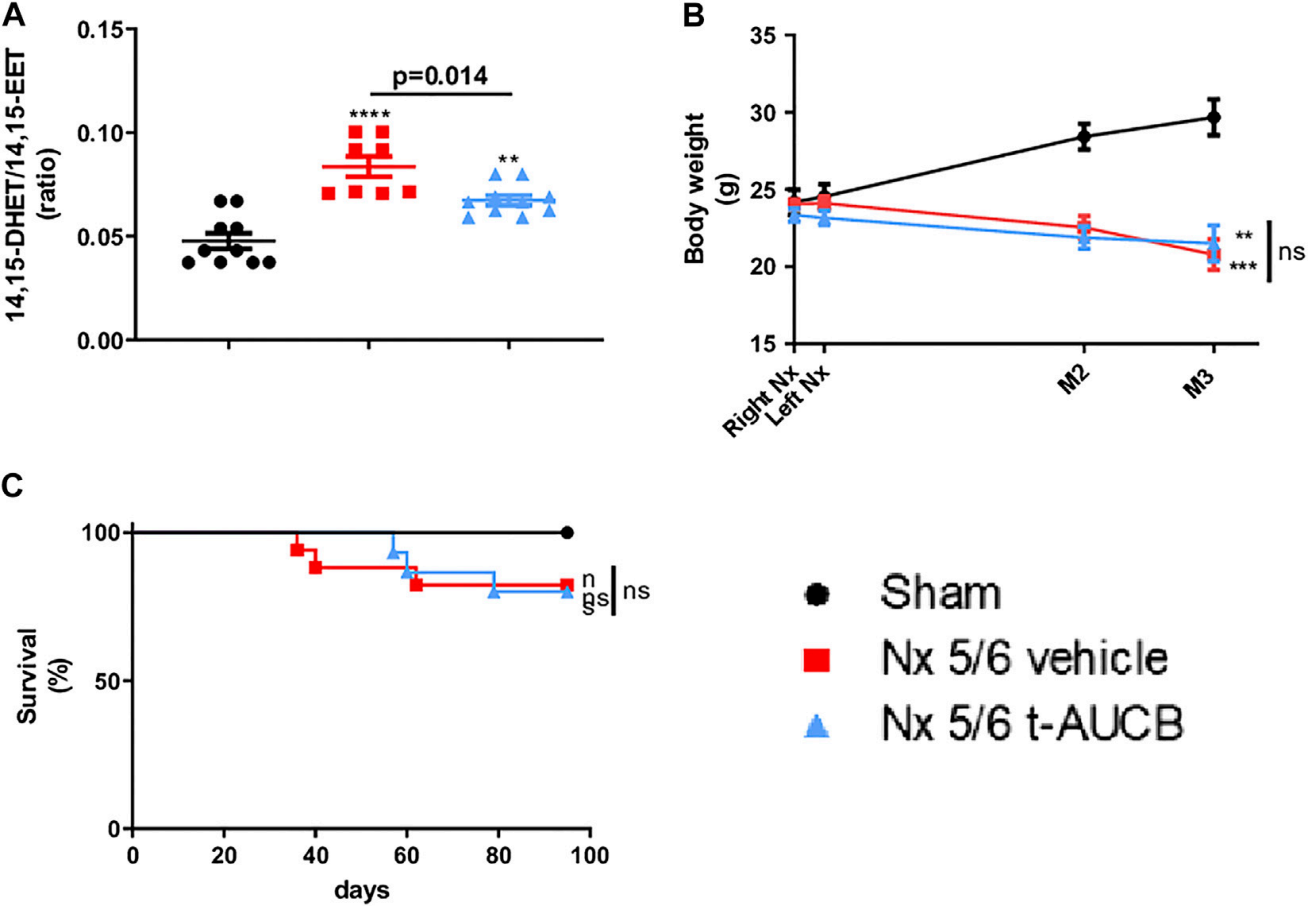
Plasma concentration 14,15-DHET to 14,15-EET ratio **(A)** at sacrifice (n = 8–10 per group). ***p < 0.001: Nx 5/6 vehicle vs. sham, **p < 0.01: Nx 5/6 *t*-AUCB vs. sham. Body weight evolution **(C)** from right Nx (month 0) to month 3 (*n* = 12–14 per group). ***p < 0.001: Nx 5/6 vehicle vs. sham, **p < 0.01: Nx 5/6 *t*-AUCB vs. sham. Survival **(D)** (*n* = 12–17 per group).

Compared to sham-operated mice, we observed an increase in 14,15-DHET-to-14,15-EET ratio in 5/6 Nx mice treated with the vehicle (Figures 1A,B), demonstrating increased sEH activity. As expected, this increase was reduced by the sEH inhibitor *t*-AUCB. The 11,12-DHET-to-11,12-EET ratio followed the same trend as 14,15-DHET-to-14,15-EET ratio but the effects was less marked and that there was no change in 8,9-DHET-to-8,9-EET. In addition, 5/6 Nx was associated with an increase in HETE plasma levels that was prevented by *t*-AUCB (Supplementary Figures S1A–C).

### Body Weight, Survival

Body weight increased in sham-operated mice from surgery to sacrifice but not in 5/6 Nx mice treated with either vehicle or *t*-AUCB (Figure 1B), without significant change in survival between groups. (Figure 1C).

### Kidney Parameters

**FIGURE 2:**
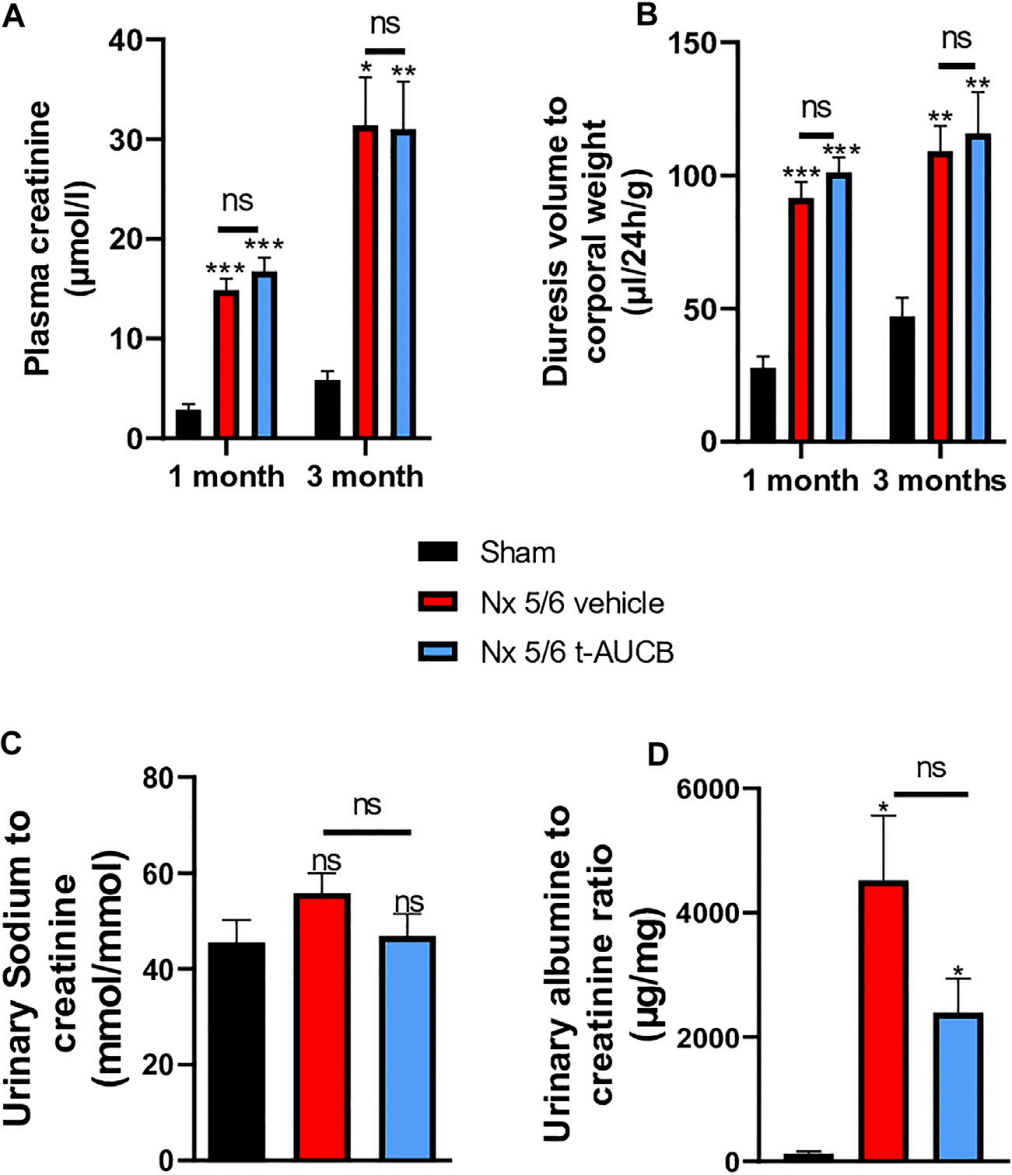
Evolution of plasma **(A)** creatinine 1 and 3 months after 5/6 Nx (n = 7–12 per group). ***p < 0.001: Nx 5/6 vehicle vs. Sham, ***p < 0.001: Nx 5/6 *t*-AUCB vs. Sham, *p < 0.05: Nx 5/6 vehicle vs. Sham, **p < 0.01: Nx 5/6 *t*-AUCB vs. Sham, ns: not-significant. Diuresis evolution **(B)** 1 and 3 months after Nx 5/6 (n = 8–12 per group). ***p < 0.001: Nx 5/6 vehicle vs. Sham, ***p < 0.001: Nx 5/6 *t*-AUCB vs. Sham, **p < 0.01: Nx 5/6 vehicle vs. Sham, **p < 0.01: Nx 5/6 *t*-AUCB vs. Sham, ns: not-significant. Comparison of urinary albumin to creatinine ratio **(C)** (n = 5-7 per group). *p < 0.05: Nx 5/6 vehicle vs. Sham, ***p < 0.05: Nx 5/6 *t*-AUCB vs. Sham, ns: not-significant. Urinary sodium to creatinine ratio (*n* = 8–12 per group). ns: not-significant.

**FIGURE 3:**
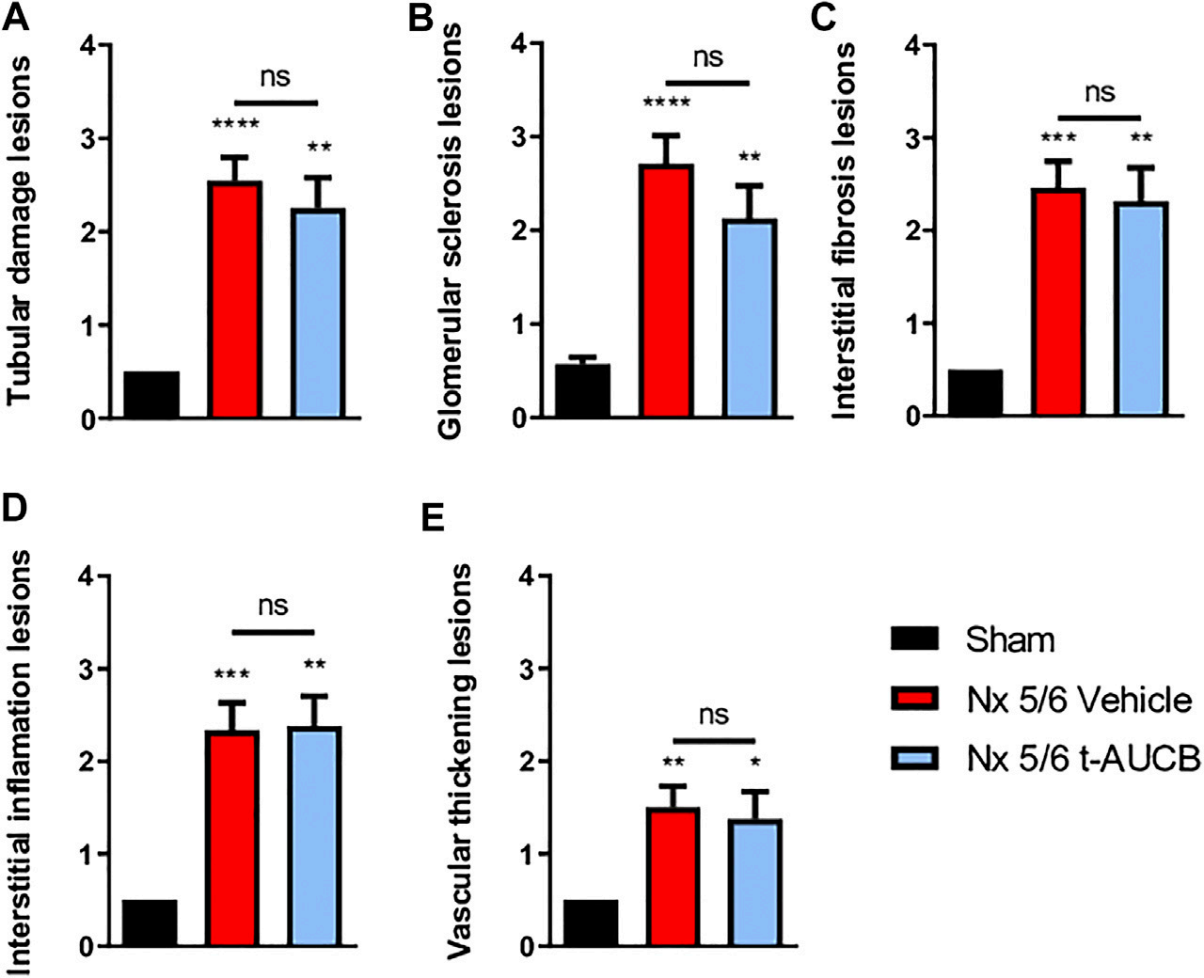
Scoring of kidney lesions at sacrifice (*n* = 7–12 per group). **(A)** Tubular damage lesions, **(B)** Glomerular sclerosis lesions, **(C)** Interstitial fibrosis lesions, **(D)** Interstitial inflammation lesions, **(E)** Vascular thickening lesions. ****p < 0.0001: Nx 5/6 vehicle vs. Sham, **p < 0.01: Nx 5/6 *t*-AUCB vs. Sham, ***p < 0.001: Nx 5/6 vehicle vs. Sham, **p < 0.01: Nx 5/6 vehicle vs. Sham, *p < 0.05: Nx 5/6 *t*-AUCB vs. Sham, ns: not-significant.

The onset of CKD was demonstrated by a significant increase in plasma creatinine 1 and 3 months after 5/6 Nx (Figure 2A) which was not prevented by *t*-AUCB. In addition, the urine volume 1 and 3 months after surgery and the urine albumin/creatinine ratio at 3 months increased to a similar extent in 5/6 Nx mice treated with vehicle or *t*-AUCB (Figures 2B,C). Similarly, the development of glomerulosclerosis, interstitial fibrosis, inflammation, tubular injury and vascular lesions induced by 5/6 Nx was not modified by *t*-AUCB (Figures 3A–E, Supplementary Figure S3).

### Systemic Hemodynamics and Cardiac Parameters

**FIGURE 4:**
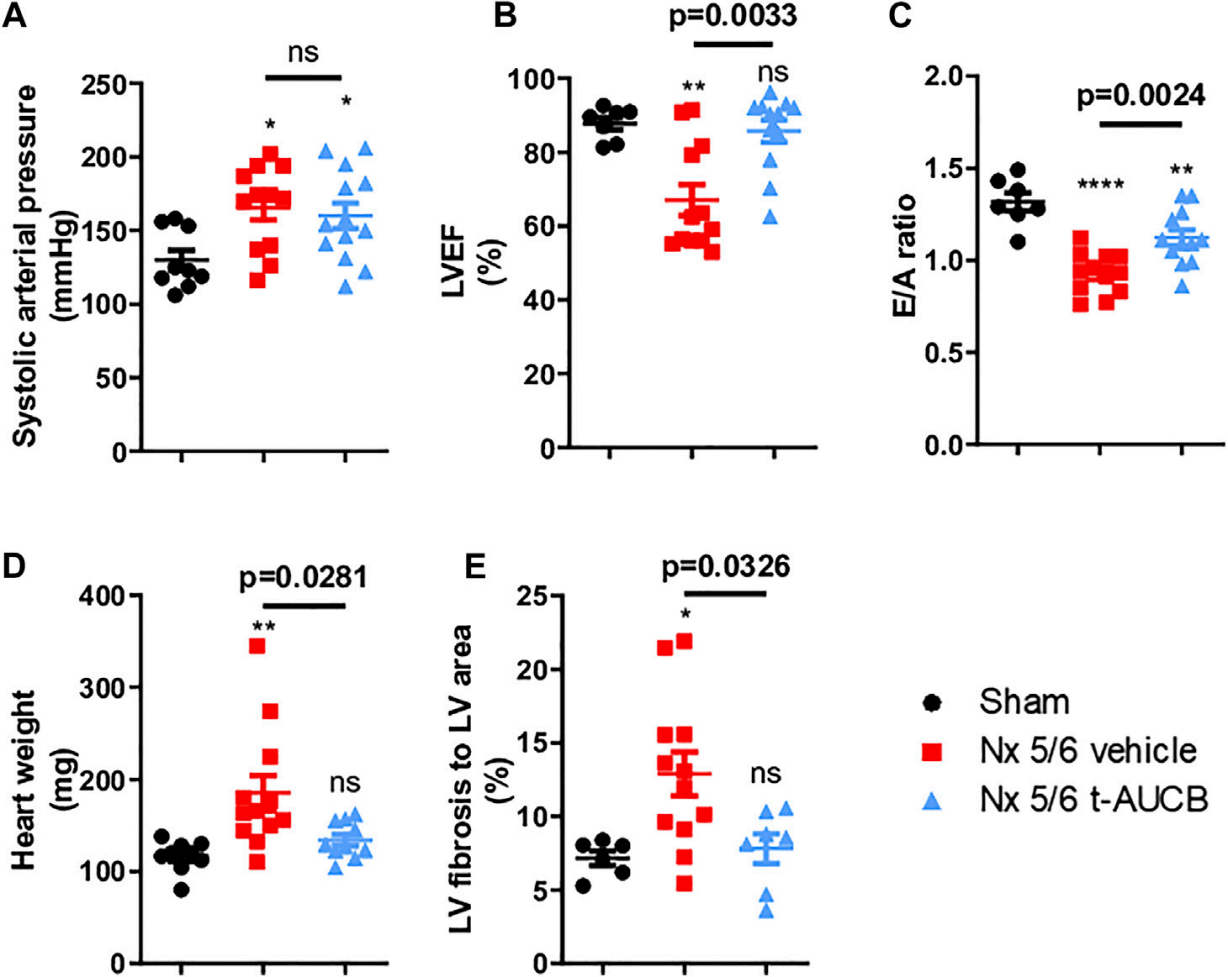
Systolic arterial pressure **(A)** 3 months after surgery (n = 9–12 per group). *p < 0.05: Nx 5/6 vehicle vs. Sham, *p < 0.05: Nx 5/6 *t*-AUCB vs. Sham, ns: not-significant. Systolic cardiac function **(B)** evaluated by left ventricular ejection fraction (LVEF) in echocardiography (*n* = 7–12 per group). **p < 0.01: Nx 5/6 vehicle vs. Sham, ns: not-significant Nx 5/6 *t*-AUCB vs. Sham. Diastolic cardiac function **(C)** evaluated by E to A ratio measured in echocardiography (*n* = 7–12 per group). ****p < 0.0001: Nx 5/6 vehicle vs. Sham, **p < 0.01: Nx 5/6 *t*-AUCB vs. Sham. Comparison in heart weight after sacrifice **(D)** (*n* = 9–12 per group). **p < 0.01: Nx 5/6 vehicle vs. Sham, ns: not-significant Nx 5/6 *t*-AUCB vs. Sham. Comparison of left ventricular (LV) fibrosis area to LV area (*n* = 7–11 per group). *p < 0.05: Nx 5/6 vehicle vs. Sham, ns: not-significant Nx 5/6 *t*-AUCB vs. Sham.

Compared to sham-operated mice, 5/6 Nx mice treated with vehicle or *t*-AUCB followed a similar pattern of increase in systolic arterial pressure after surgery. While systolic arterial pressure was not significantly increased after 1 month, both groups similarly presented a significant increase at 3 months post 5/6 Nx (Figure 4A). One month after 5/6 Nx LVEF and E/A ratio were non-significantly altered, while both parameters decreased significantly 3 months after 5/6 Nx in mice treated with vehicle but not in mice treated with *t*-AUCB (Figures 4B,C). In addition, *t*-AUCB prevented the increase in heart weight as well as LV fibrosis induced by 5/6 Nx (Figures 4D,E and Supplementary Figure S3). This was observed while *t*-AUCB was able to prevent the increase in urinary aldosterone, but not in plasma FGF-23 nor heart expression of ICAM-1 and VCAM-1 mRNA (Supplementary Figures S2A,B).

### Vascular Parameters

**FIGURE 5:**
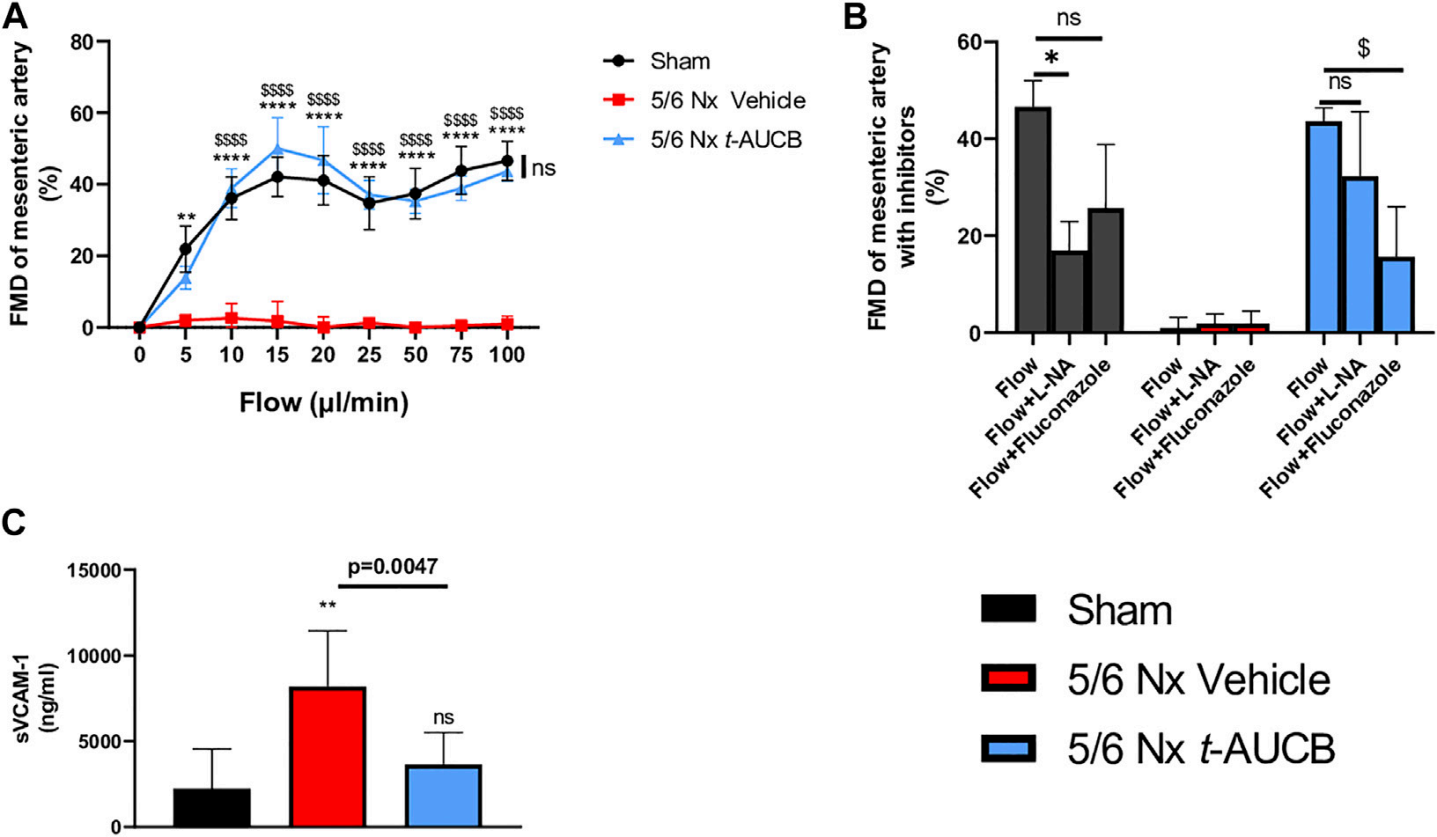
Flow mediated dilatation (FMD) of second mesenteric resistance artery **(A)** at sacrifice (*n* = 5-8 per group). **p < 0.01: Nx 5/6 vehicle vs. Sham, ****p < 0.0001: Nx 5/6 vehicle vs. Sham, $$$$ *p* < 0.0001 Nx 5/6 vehicle vs. Nx 5/6 *t*-AUCB, ns: not-significant Nx 5/6 *t*-AUCB vs. Sham. FMD in basal condition and after inhibitors (L-NA and Fluconazole) at flow 100 μL/min (*n* = 5-8 per group). *p < 0.05 flow vs. Flow + L-NA, $ *p* < 0.05 flow vs. Flow + Fluconazole. ns: not-significant. Plasma soluble VCAM1 concentration **(C)** at sacrifice (*n* = 5–10 per group). **p < 0.01: Nx 5/6 vehicle vs. Sham, ns: not-significant Nx 5/6 *t*-AUCB vs. Sham.

FMD was abolished in 5/6 Nx mice treated with vehicle and fully prevented by *t*-AUCB (Figure 5A). FMD was decreased by l-NAME in sham-operated mice without significant effect of fluconazole infusion (Figure 5B), demonstrating a predominant role of NO in this response. However, FMD restoration in 5/6 Nx mice treated with *t*-AUCB was mainly due to the potentiation of CYP450-dependent pathway, as shown by the significant decrease induced by the CYP450 epoxygenases inhibitor fluconazole but not by l-NAME. In agreement with the improvement in endothelial function, *t*-AUCB prevented the increase in plasma sVCAM-1 (Figure 5C).

## Discussion

This study provides the first demonstration that pharmacological inhibition of sEH prevents the development of cardiac and vascular disorders due to CKD, independently of kidney function.

The experimental model of 5/6 Nx used here led to a significant and progressive decrease in kidney function, with increased albuminuria and chronic kidney lesions on histology. As is the case in human CKD, this was associated with the onset of hypertension, diastolic and mild systolic dysfunctions, heart hypertrophy, and endothelial dysfunction. These results are in accordance with previously published studies in rodent models of subtotal nephrectomy and provide a reliable experimental setting to analyse the CV consequences of CKD (Ma and Fogo, 2003; Siedlecki et al., 2009; Winterberg et al., 2016; Neuburg et al., 2018; Hamzaoui et al., 2020).

5/6 Nx was associated with an impaired arachidonic acid metabolism, characterized by an increase in 14,15-DHET-to-14,15-EET, and of pro-inflammatory HETEs. Since EETs have potent vasodilator, anti-inflammatory and anti-fibrotic properties, we hypothesized that promoting a more physiological balance with a pharmacological inhibitor of sEH, the key enzyme of EETs degradation, may have beneficial effects on both the heart and the vessels in this setting. As expected, the administration of *t*-AUCB prevented the increase in the DHETs/EETs ratio in 5/6 Nx mice.

Importantly, *t*-AUCB administered for 3 months did not modify the kidney consequences of 5/6 Nx. 5/6 Nx mice treated with *t*-AUCB presented a similar increase in plasma creatinine, albuminuria and kidney lesions compared to mice treated with vehicle. These results were in accordance with a previous study by Jung O et al (Jung et al., 2010), where no difference in kidney histological damage was observed when mice were treated by *c*-AUCB, the *cis*-isomer of *t*-AUCB which possesses similar potency against sEH activity, during 8 weeks. 5/6 Nx mice treated with *t*-AUCB showed no difference in urine output when compared to vehicle, and no difference in sodium excretion. In contrast, previous studies have reported an increase in sodium excretion, related to a decreased reabsorption of sodium as the consequence of tubular effects of EETs (Pavlov et al., 2011; Wang Q. et al., 2013; Wang et al., 2014; Schragenheim et al., 2018). The discrepancy with our results could be explained by the predominant impact of severely decreased glomerular filtration rate, reducing the tubular effects of EETs. After 5/6 Nx, no difference in albuminuria was observed between mice treated with *t*-AUCB and vehicle. On the contrary, Jung O et al (Jung et al., 2010) reported increased albuminuria with *c*-AUCB. This difference could be explained by the longer duration of treatment in our study (8 weeks vs. 3 months) that could allow normalization of arachidonic acid metabolism imbalance induced by short-term inhibition of sEH*.* In fact, in the previous study (Jung et al., 2010), sEH inhibition was shown to potentiate the formation of pro-inflammatory HETEs while in our study, HETEs were reduced by 3 month *t*-AUCB administration*.* In addition, given that 14,15-DHET-to-14,15-EET remained elevated compared to sham mice, we could hypothesize that only a partial blockade of sEH occurs, allowing to prevent the shift of arachidonic acid from the CYP450 pathway into the LOX pathway. Accordingly, studies performed in hyperglycaemic overweight mice and mice fed a high-fat diet (Roche et al., 2015b; Luo et al., 2019) even suggest beneficial effects of *t*-AUCB on albuminuria and kidney lesions.

The analysis of direct effects of treatments on CV complications associated with CKD is frequently limited by a parallel impact of the treatment on the kidney disease. In the present study the fact that *t*-AUCB had no significant impact on kidney function and lesions after 5/6 Nx provides an ideal setting to investigate the direct CV impact of sEH inhibition in type 4 cardiorenal syndrome.

Mice treated with *t*-AUCB showed improving systolic and diastolic heart function induced by 5/6 Nx when compared with vehicle. In addition, the increase in heart weight and the onset of LV fibrosis were blunted by *t*-AUCB. In CKD, progressive diffuse myocardial fibrosis and LV hypertrophy contribute to impair the relaxation of the cardiac wall (Zannad Faiez and Rossignol Patrick, 2018). sEH inhibition prevents cardiac hypertrophy in experimental models based on angiotensin II infusion (Ai et al., 2009), aortic banding (Xu et al., 2006), renovascular hypertension (two kidney one clip) (Gao et al., 2011; Wang Z. H. et al., 2013) and metabolic disease related to obesity and insulin resistance (Roche et al., 2015a). This preventive effect was associated with a reduction of myocardial fibrosis, as observed in our study. Experimental and clinical evidences point out a role of FGF-23 in CKD-associated cardiac hypertrophy since FGF-23 increases in patients with CKD and its concentration is correlated to cardiac hypertrophy (Faul et al., 2011; Isakova et al., 2011). In our study, plasma FGF-23 increased after 5/6 Nx without normalization by *t*-AUCB. This suggests that the beneficial effect of *t*-AUCB on the heart is independent of FGF-23. Cardiac hypertrophy can be an adaptive response to the increased afterload related to arterial hypertension. Inhibition of sEH reduced blood pressure in several studies in mice and rats (Yu et al., 2000; Huang et al., 2007; Gao et al., 2011) but *t*-AUCB had no significant effect on the increase of systolic blood pressure after 5/6 Nx, excluding blood pressure as the mediator of *t*-AUCB beneficial effects in this setting. Similarly, Jung O *et al* (Jung et al., 2010) reported no reduction of hypertension with an inhibitor of sEH after subtotal nephrectomy. In fact, the anti-hypertensive effect of sEH inhibition is usually only observed in angiotensin II-dependent hypertension models, which is only partially the case in this setting (Jung et al., 2005).

**FIGURE 6:**
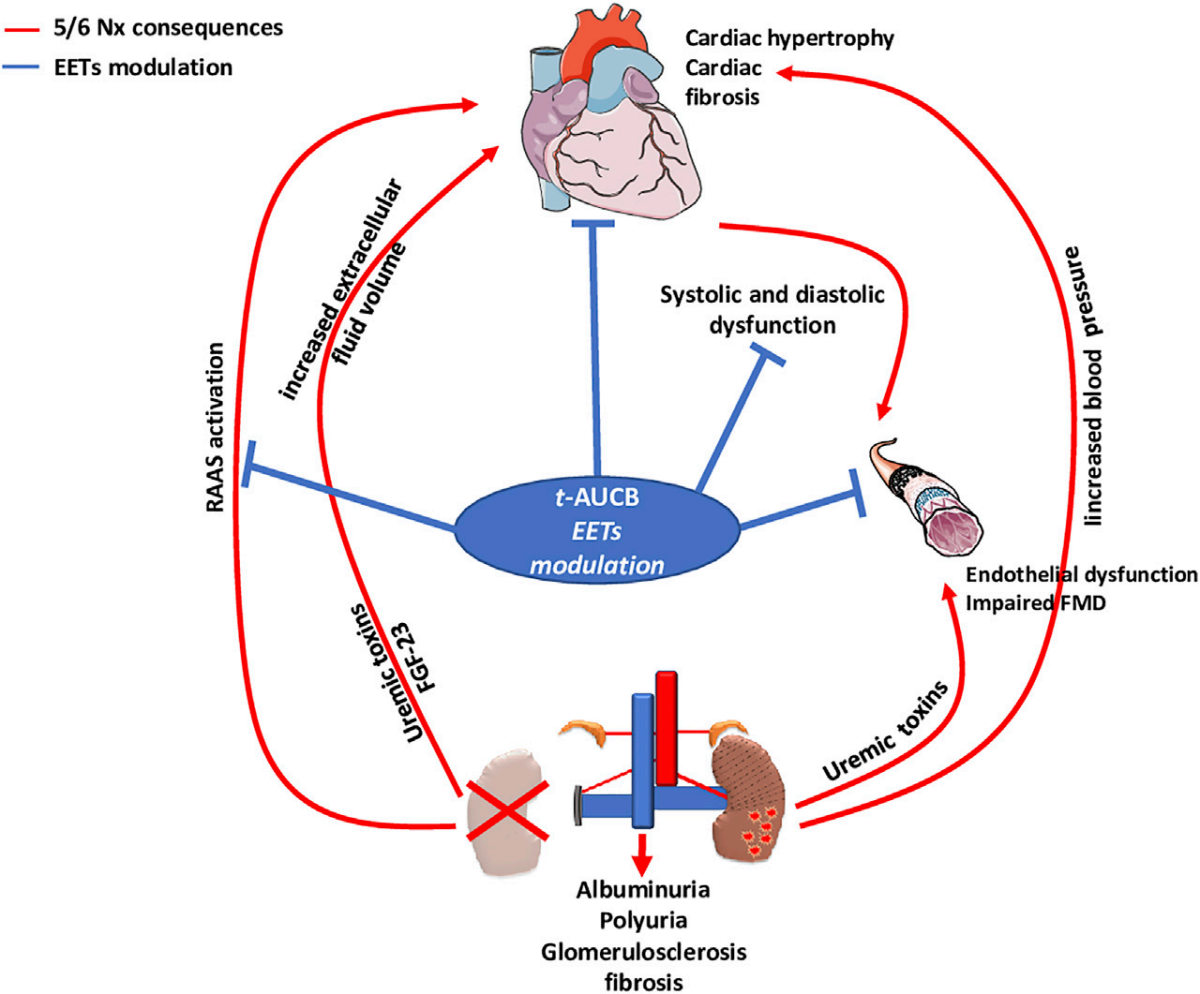
Type 4 cardio-renal syndrome and impact of EETs modulation by *t*-AUCB.

FMD of resistance arteries is a gold-standard technique to measure the endothelial function in experimental settings. FMD was fully abolished after 5/6 Nx in mice treated with vehicle but was not affected in Nx mice treated with t-AUCB compared to sham-operated mice. Complete dilatation of the mesenteric artery after incubation with SNP, a donor of NO, was observed in all groups showing normal smooth muscle cell function. Together, these results clearly demonstrate that the endothelial dysfunction associated with CKD in our study was prevented by sEH inhibition. Moreover, the improved FMD observed with *t*-AUCB was predominantly mediated by EETs since *ex vivo* inhibition of this pathway with fluconazole blunted FMD. Furthermore, plasma soluble VCAM-1, a surrogate marker for endothelial dysfunction, was sharply increased after 5/6 Nx and *t*-AUCB prevented this increase. Accordingly, similar improvement in endothelial function associated with inhibition of sEH has been observed in animal models of hypertension and insulin resistance (Gao et al., 2011; Zhang et al., 2011; Roche et al., 2015a). In CKD, endothelial dysfunction is multifactorial and considered as an important mediator of CV diseases (Jourde-Chiche et al., 2019). At the organ level, functional and structural alterations of endothelial cells promote inflammation, hypoxia and mesenchymal transition, which are putative mechanisms of cardiac fibrosis in CKD (Yu et al., 2000; Zannad Faiez and Rossignol Patrick, 2018). Together, the protection against microvascular endothelial dysfunction could be a mediator of the cardiac beneficial effects elicited by *t*-AUCB after 5/6 Nx. EETs modulation effects observed in our study were summarized in Figure 6.

## Conclusion

This study provides the first evidence that inhibiting sEH prevents the endothelial dysfunction and cardiac consequences associated with experimental CKD. These beneficial CV effects were independent of both kidney function and hypertension. Preventing EETs degradation by the blockade of sEH therefore appears as a novel and relevant target for the long-term management of type 4 cardiorenal syndrome.

## Data Availability

The original contributions presented in the study are included in the article/Supplementary Material, further inquiries can be directed to the corresponding author.

## References

[B1] Ai D. Pang W. Li N. Xu M. Jones P. D. Yang J. (2009). Soluble epoxide hydrolase plays an essential role in angiotensin II-induced cardiac hypertrophy. Proc. Natl. Acad. Sci. U.S.A. 106, 564–569. 10.1073/pnas.0811022106 19126686 PMC2626743

[B2] Charytan D. M. Fishbane S. Malyszko J. McCullough P. A. Goldsmith D. (2015). Cardiorenal syndrome and the role of the bone-mineral axis and anemia. Am. J. Kidney Dis. 66, 196–205. 10.1053/j.ajkd.2014.12.016 25727384 PMC4516683

[B3] Duflot T. Roche C. Lamoureux F. Guerrot D. Bellien J. (2014). Design and discovery of soluble epoxide hydrolase inhibitors for the treatment of cardiovascular diseases. Expert Opin. Drug Discov. 9, 229–243. 10.1517/17460441.2014.881354 24490654

[B4] Duflot T. Moreau-Grangé L. Roche C. Iacob M. Wils J. Rémy-Jouet I. (2019). Altered bioavailability of epoxyeicosatrienoic acids is associated with conduit artery endothelial dysfunction in type 2 diabetic patients. Cardiovasc. Diabetol. 18, 35. 10.1186/s12933-019-0843-z 30885203 PMC6423843

[B5] Duflot T. Pereira T. Roche C. Iacob M. Cardinael P. Hamza N. E. (2017). A sensitive LC-MS/MS method for the quantification of regioisomers of epoxyeicosatrienoic and dihydroxyeicosatrienoic acids in human plasma during endothelial stimulation. Anal. Bioanal. Chem. 409, 1845–1855. 10.1007/s00216-016-0129-1 27981341

[B6] Faul C. Amaral A. P. Oskouei B. Hu M. C. Sloan A. Isakova T. (2011). FGF23 induces left ventricular hypertrophy. J. Clin. Invest. 121, 4393–4408. 10.1172/JCI46122 21985788 PMC3204831

[B7] Gao J. Bellien J. Gomez E. Henry J. P. Dautreaux B. Bounoure F. (2011). Soluble epoxide hydrolase inhibition prevents coronary endothelial dysfunction in mice with renovascular hypertension. J. Hypertens. 29, 1128–1135. 10.1097/HJH.0b013e328345ef7b 21451419

[B8] Guerrot D. Kerroch M. Placier S. Vandermeersch S. Trivin C. Mael-Ainin M. (2011). Discoidin domain receptor 1 is a major mediator of inflammation and fibrosis in obstructive nephropathy. Am. J. Pathol. 179, 83–91. 10.1016/j.ajpath.2011.03.023 21640971 PMC3123843

[B9] Hamzaoui M. Djerada Z. Brunel V. Mulder P. Richard V. Bellien J. (2020). 5/6 nephrectomy induces different renal, cardiac and vascular consequences in 129/Sv and C57BL/6JRj mice. Sci. Rep. 10, 1524–1529. 10.1038/s41598-020-58393-w 32001792 PMC6992698

[B10] Hatamizadeh P. Fonarow G. C. Budoff M. J. Darabian S. Kovesdy C. P. Kalantar-Zadeh K. (2013). Cardiorenal syndrome: Pathophysiology and potential targets for clinical management. Nat. Rev. Nephrol. 9, 99–111. 10.1038/nrneph.2012.279 23247571

[B11] Hill N. R. Fatoba S. T. Oke J. L. Hirst J. A. O’Callaghan C. A. Lasserson D. S. (2016). Global prevalence of chronic kidney disease - a systematic review and meta-analysis. PLoS One 11, e0158765. 10.1371/journal.pone.0158765 27383068 PMC4934905

[B12] Huang H. Morisseau C. Wang J. Yang T. Falck J. R. Hammock B. D. (2007). Increasing or stabilizing renal epoxyeicosatrienoic acid production attenuates abnormal renal function and hypertension in obese rats. Am. J. Physiol. Ren. Physiol. 293, F342–F349. 10.1152/ajprenal.00004.2007 17442729

[B13] Imig J. D. Zhao X. Capdevila J. H. Morisseau C. Hammock B. D. (2002). Soluble epoxide hydrolase inhibition lowers arterial blood pressure in angiotensin II hypertension. Hypertension 39, 690–694. 10.1161/hy0202.103788 11882632

[B14] Isakova T. Wahl P. Vargas G. S. Gutiérrez O. M. Scialla J. Xie H. (2011). Fibroblast growth factor 23 is elevated before parathyroid hormone and phosphate in chronic kidney disease. Kidney Int. 79, 1370–1378. 10.1038/ki.2011.47 21389978 PMC3134393

[B15] Jourde-Chiche N. Fakhouri F. Dou L. Bellien J. Burtey S. Frimat M. (2019). Endothelium structure and function in kidney health and disease. Nat. Rev. Nephrol. 15, 87–108. 10.1038/s41581-018-0098-z 30607032

[B16] Jung O. Brandes R. P. Kim I. H. Schweda F. Schmidt R. Hammock B. D. (2005). Soluble epoxide hydrolase is a main effector of angiotensin II-induced hypertension. Hypertension 45, 759–765. 10.1161/01.HYP.0000153792.29478.1d 15699457

[B17] Jung O. Jansen F. Mieth A. Barbosa-Sicard E. Pliquett R. U. Babelova A. (2010). Inhibition of the soluble epoxide hydrolase promotes albuminuria in mice with progressive renal disease. PLoS One 5, e11979. 10.1371/journal.pone.0011979 20694143 PMC2915917

[B18] Kovesdy C. P. Quarles L. D. (2013). Fibroblast growth factor-23: what we know, what we don't know, and what we need to know. Nephrol. Dial. Transpl. 28, 2228–2236. 10.1093/ndt/gft065 PMC3769978 23625971

[B19] Kovesdy C. P. Quarles L. D. (2016). FGF23 from bench to bedside. Am. J. Physiol. Ren. Physiol. 310, F1168–F1174. 10.1152/ajprenal.00606.2015 PMC6425620 26864938

[B20] Lee C. R. North K. E. Bray M. S. Fornage M. Seubert J. M. Newman J. W. (2006). Genetic variation in soluble epoxide hydrolase (EPHX2) and risk of coronary heart disease: the atherosclerosis risk in communities (ARIC) study. Hum. Mol. Genet. 15, 1640–1649. 10.1093/hmg/ddl085 16595607 PMC2040335

[B21] Lee C. R. Pretorius M. Schuck R. N. Burch L. H. Bartlett J. Williams S. M. (2011). Genetic variation in soluble epoxide hydrolase (EPHX2) is associated with forearm vasodilator responses in humans. Hypertension 57, 116–122. 10.1161/HYPERTENSIONAHA.110.161695 21098312 PMC3020911

[B22] Liu J. Y. (2018). Inhibition of soluble epoxide hydrolase for renal health. Front Pharmacol. 9, 1551. 10.3389/fphar.2018.01551 30687105 PMC6335332

[B23] Luo Y. Wu M. Y. Deng B. Q. Huang J. Hwang S. H. Li M. Y. (2019). Inhibition of soluble epoxide hydrolase attenuates a high-fat diet-mediated renal injury by activating PAX2 and AMPK. Proc. Natl. Acad. Sci. U S A. 116, 5154–5159. 10.1073/pnas.1815746116 30804206 PMC6421466

[B24] Ma L. J. Fogo A. B. (2003). Model of robust induction of glomerulosclerosis in mice: importance of genetic background. Kidney Int. 64, 350–355. 10.1046/j.1523-1755.2003.00058.x 12787428

[B25] Merabet N. Bellien J. Glevarec E. Nicol L. Lucas D. Remy-Jouet I. (2012). Soluble epoxide hydrolase inhibition improves myocardial perfusion and function in experimental heart failure. J. Mol. Cel. Cardiol. 52, 660–666. 10.1016/j.yjmcc.2011.11.015 22155238

[B26] Neuburg S. Dussold C. Gerber C. Wang X. Francis C. Qi L. (2018). Genetic background influences cardiac phenotype in murine chronic kidney disease. Nephrol. Dial. Transplant. 33, 1129–1137. 10.1093/ndt/gfx332 29309658 PMC6030849

[B27] Pavlov T. S. Ilatovskaya D. V. Levchenko V. Mattson D. L. Roman R. J. Staruschenko A. (2011). Effects of cytochrome P-450 metabolites of arachidonic acid on the epithelial sodium channel (ENaC). Am. J. Physiol. Ren. Physiol. 301, F672–F681. 10.1152/ajprenal.00597.2010 PMC3174560 21697242

[B28] Rangaswami J. Bhalla V. Blair J. E. A. Chang T. I. Costa S. Lentine K. L. (2019). Cardiorenal syndrome: classification, pathophysiology, diagnosis, and treatment strategies: A scientific statement from the American heart association. Circulation 139, e840–e878. 10.1161/CIR.0000000000000664 30852913

[B29] Roche C. Besnier M. Cassel R. Harouki N. Coquerel D. Guerrot D. (2015a). Soluble epoxide hydrolase inhibition improves coronary endothelial function and prevents the development of cardiac alterations in obese insulin-resistant mice. Am. J. Physiol. Heart Circ. Physiol. 308, H1020–H1029. 10.1152/ajpheart.00465.2014 25724490 PMC4551118

[B30] Roche C. Guerrot D. Harouki N. Duflot T. Besnier M. Rémy-Jouet I. (2015b). Impact of soluble epoxide hydrolase inhibition on early kidney damage in hyperglycemic overweight mice. Prostaglandins Other Lipid Mediat. 120, 148–154. 10.1016/j.prostaglandins.2015.04.011 26022136 PMC4575616

[B31] Schragenheim J. Bellner L. Cao J. Singh SP. Bamshad D. McClung JA. (2018). EET enhances renal function in obese mice resulting in restoration of HO-1-Mfn1/2 signaling, and decrease in hypertension through inhibition of sodium chloride co-transporter. Prostaglandins Other Lipid Mediat. 137, 30–39. 10.1016/j.prostaglandins.2018.05.008 29787809 PMC6075657

[B32] Scialla J. J. Xie H. Rahman M. Anderson A. H. Isakova T. Ojo A. (2014). Fibroblast growth factor-23 and cardiovascular events in CKD. J. Am. Soc. Nephrol. 25, 349–360. 10.1681/ASN.2013050465 24158986 PMC3904568

[B33] Siedlecki A. M. Jin X. Muslin A. J. (2009). Uremic cardiac hypertrophy is reversed by rapamycin but not by lowering of blood pressure. Kidney Int. 75, 800–808. 10.1038/ki.2008.690 19165175 PMC2764402

[B34] Ter Maaten J. M. Damman K. Verhaar M. C. Paulus W. J. Duncker D. J. Cheng C. (2016). Connecting heart failure with preserved ejection fraction and renal dysfunction: the role of endothelial dysfunction and inflammation. Eur. J. Heart Fail. 18, 588–598. 10.1002/ejhf.497 26861140

[B35] Wang Q. Pang W. Cui Z. Shi J. Liu Y. Liu B. (2013). Upregulation of soluble epoxide hydrolase in proximal tubular cells mediated proteinuria-induced renal damage. Am. J. Physiol. Ren. Physiol. 304, F168–F176. 10.1152/ajprenal.00129.2012 PMC3543623 23152298

[B36] Wang Z. H. Davis B. B. Jiang D. Q. Zhao T. T. Xu D. Y. (2013). Soluble epoxide hydrolase inhibitors and cardiovascular diseases. Curr. Vasc. Pharmacol. 11, 105–111. 10.2174/157016113804547593 22303912

[B37] Wang W. H. Zhang C. Lin D. H. Wang L. Graves J. P. Zeldin D. C. (2014). Cyp2c44 epoxygenase in the collecting duct is essential for the high K+ intake-induced antihypertensive effect. Am. J. Physiol. Ren. Physiol. 307, F453–F460. 10.1152/ajprenal.00123.2014 PMC4137132 24966089

[B38] Winterberg P. D. Jiang R. Maxwell J. T. Wang B. Wagner M. B. (2016). Myocardial dysfunction occurs prior to changes in ventricular geometry in mice with chronic kidney disease (CKD). Physiol. Rep. 4, 12732. 10.14814/phy2.12732 PMC4823595 26997631

[B39] Xu D. Li N. He Y. Timofeyev V. Lu L. Tsai H. J. (2006). Prevention and reversal of cardiac hypertrophy by soluble epoxide hydrolase inhibitors. Proc. Natl. Acad. Sci. U.S.A. 103, 18733–18738. 10.1073/pnas.0609158103 17130447 PMC1693731

[B40] Yu Z. Xu F. Huse L. M. Morisseau C. Draper A. J. Newman J. W. (2000). Soluble epoxide hydrolase regulates hydrolysis of vasoactive epoxyeicosatrienoic acids. Circ. Res. 87, 992–998. 10.1161/01.res.87.11.992 11090543

[B41] Zannad F. Rossignol P. (2018). Cardiorenal syndrome revisited. Circulation 138, 929–944. 10.1161/CIRCULATIONAHA.117.028814 30354446

[B42] Zhang L. N. Vincelette J. Chen D. Gless R. D. Anandan S. K. Rubanyi G. M. (2011). Inhibition of soluble epoxide hydrolase attenuates endothelial dysfunction in animal models of diabetes, obesity and hypertension. Eur. J. Pharmacol. 654, 68–74. 10.1016/j.ejphar.2010.12.016 21187082

