# Supplementary figures and images for: Soluble Epoxide Hydrolase Inhibition Prevents Experimental Type 4 Cardiorenal Syndrome
Source: Front Mol Biosci. 2021 Mar 11;7:604042. doi: 10.3389/fmolb.2020.604042 (PMC7991096; doi:10.3389/fmolb.2020.604042)

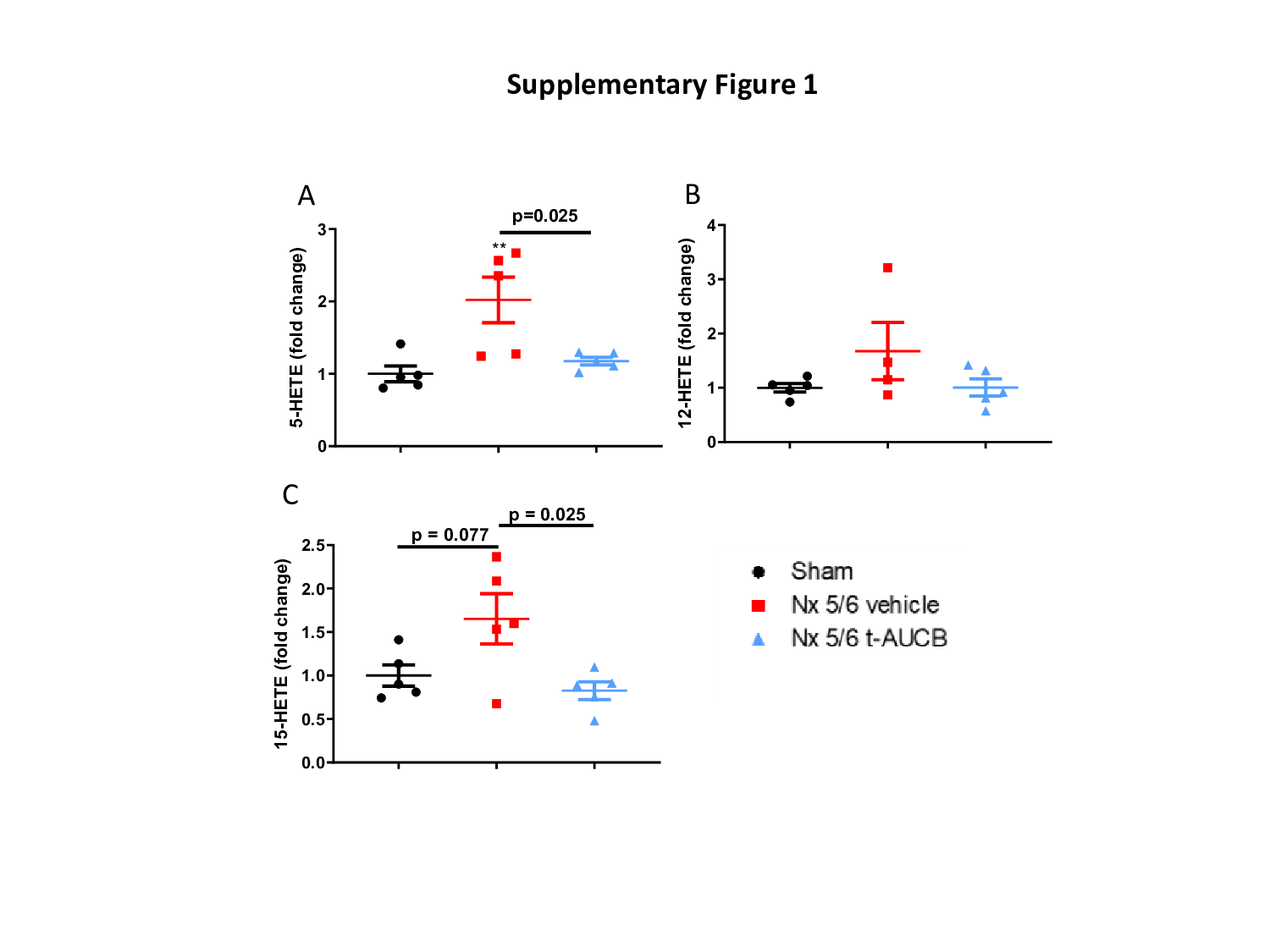

Supplement: Supplementary file 1 [file image1.tiff]

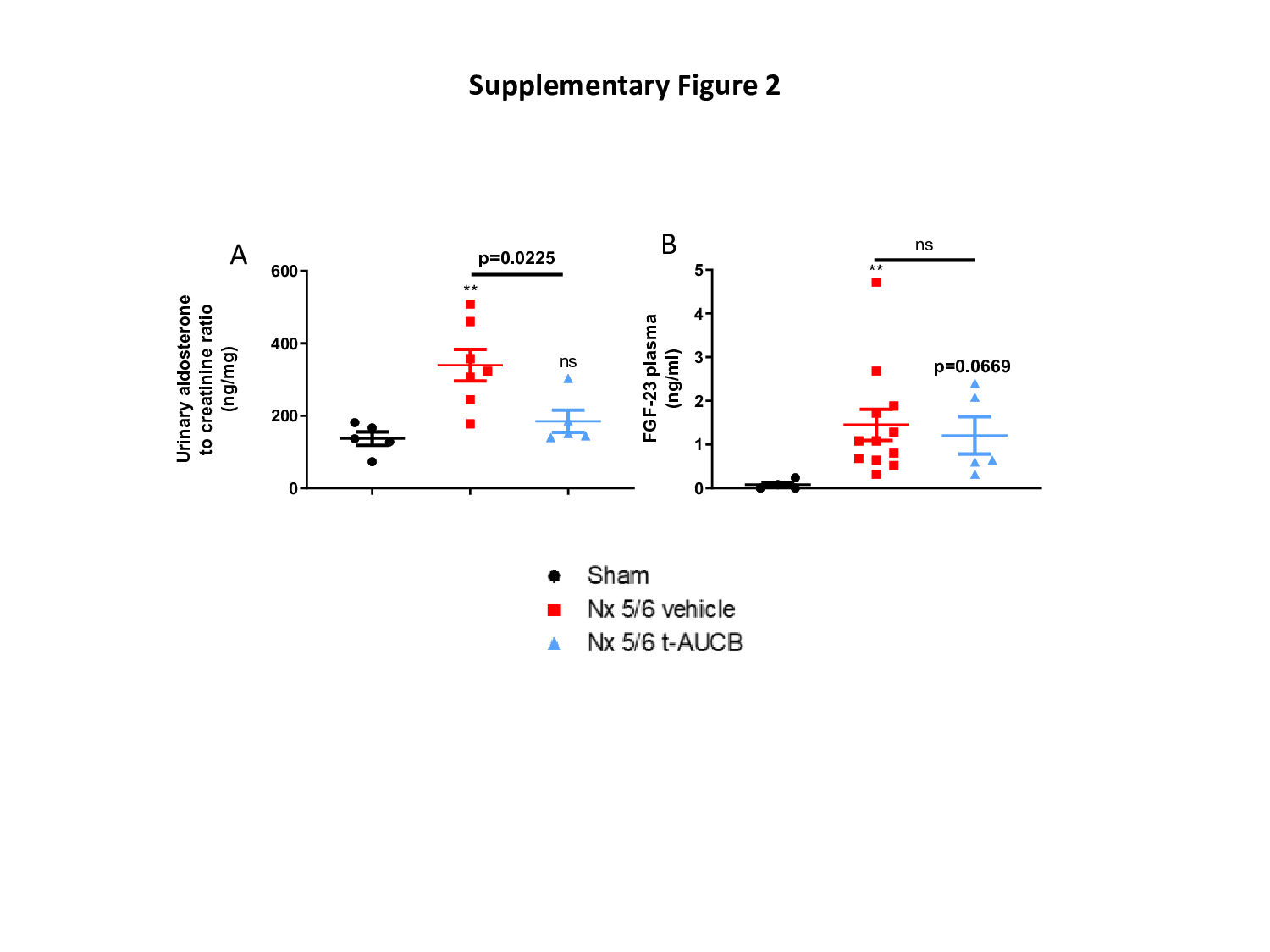

Supplement: Supplementary file 2 [file image2.tiff]

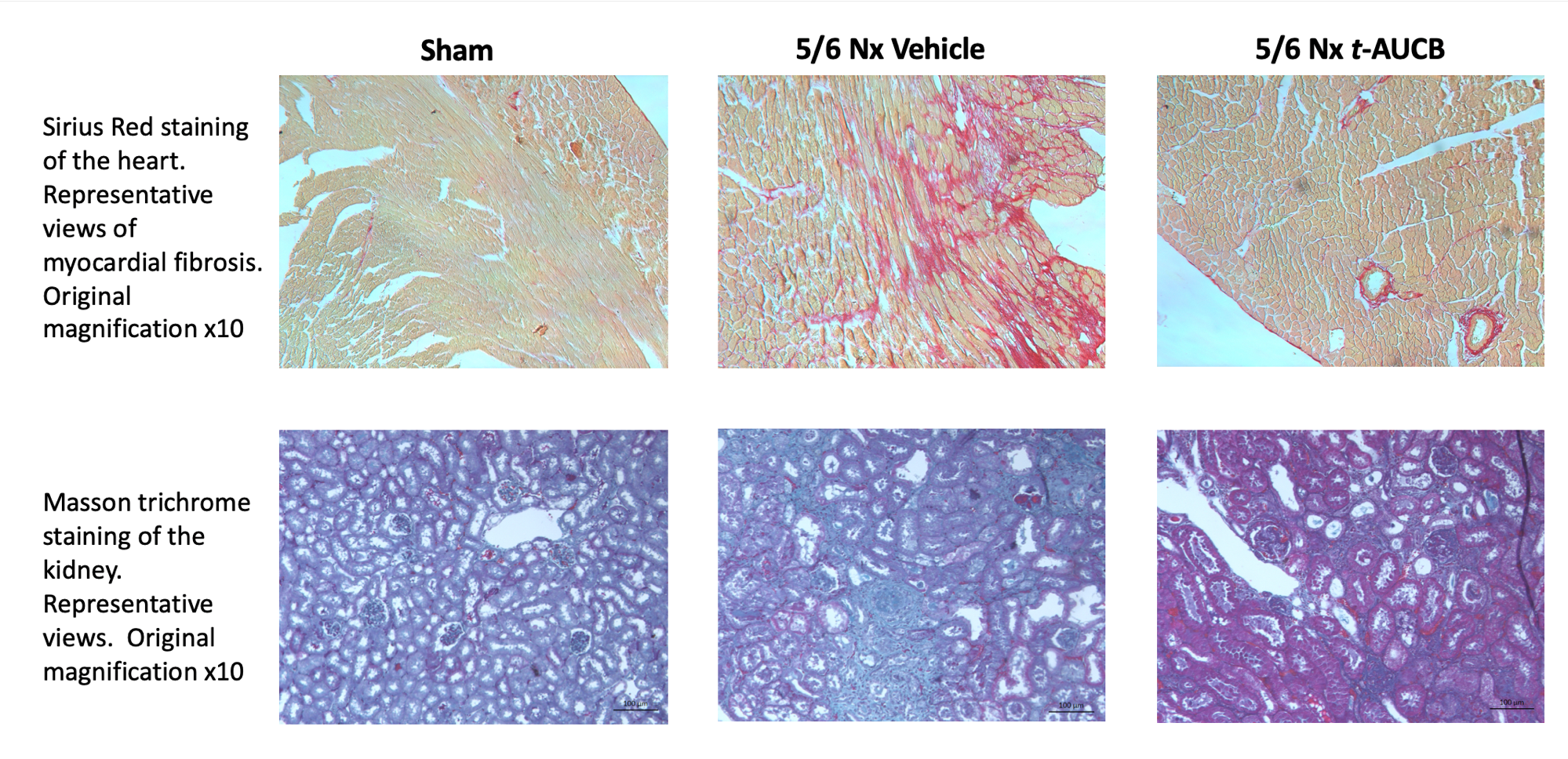

Supplement: Supplementary file 3 [file image3.tiff]
